# Supplementary material for: Inverted device architecture for high efficiency single-layer organic light-emitting diodes with imbalanced charge transport
Source: Nat Commun. 2024 May 15;15:4107. doi: 10.1038/s41467-024-48553-1 (PMC11096390; doi:10.1038/s41467-024-48553-1)
Supplement: Supplementary file 1 — Supplementary Information [file 41467_2024_48553_MOESM1_ESM.pdf]

Supplementary Information

Inverted device architecture for high efficiency single-layer organic light-emitting diodes with imbalanced charge transport

Xiao Tan[1], Dehai Dou[1], Lay-Lay Chua[2,3], Rui-Qi Png[2], Daniel G. Congrave[4], Hugo Bronstein[4,5], Martin Baumgarten[1], Yungui Li[1]*, Paul W. M. Blom[1], Gert-Jan A. H. Wetzelaer[1]*

[1]*Max Planck Institute for Polymer Research, Mainz, Germany.* [2]*Department of Physics, National University of Singapore, Singapore, Singapore.* [3]*National University of Singapore, Department of Chemistry, Lower Kent Ridge Road, Singapore S117552, Singapore.* [4]*Department of Chemistry, University of Cambridge, Cambridge, CB2 1EW, U.K.* [5]*Cavendish Laboratory, University of Cambridge, Cambridge, CB3 0HE, United Kingdom*

**yungui.li@mpip-mainz.mpg.de; wetzelaer@mpip-mainz.mpg.de*

1. Supplementary Figures

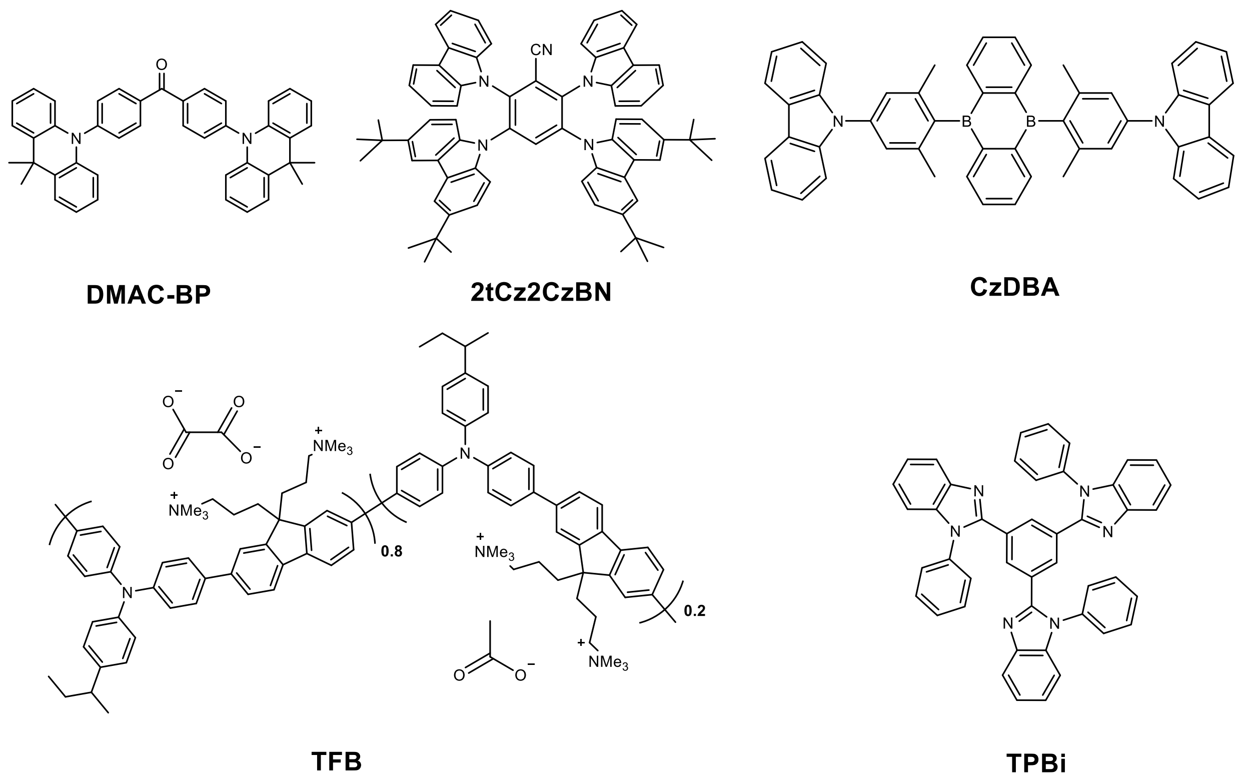

Supplementary Figure 1. Chemical structure of organic compounds used in this work.

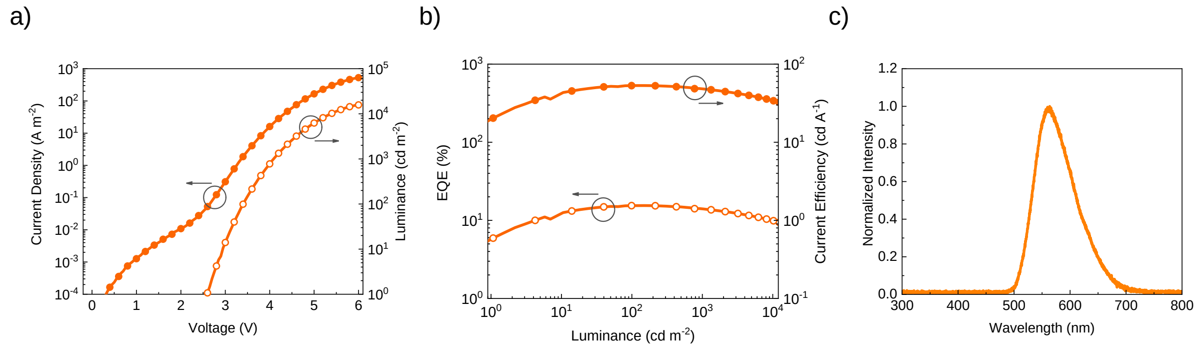

Supplementary Figure 2. Device performance of a single-layer CzDBA inverted OLED. a) Current density–voltage (close symbols) and luminance–voltage (open symbols) characteristics of a CzDBA single-layer inverted OLED. b) EQE (open symbols) and current efficiency (close symbols) as a function of luminance. c) Electroluminescence spectra of single-layer CzDBA inverted OLED.

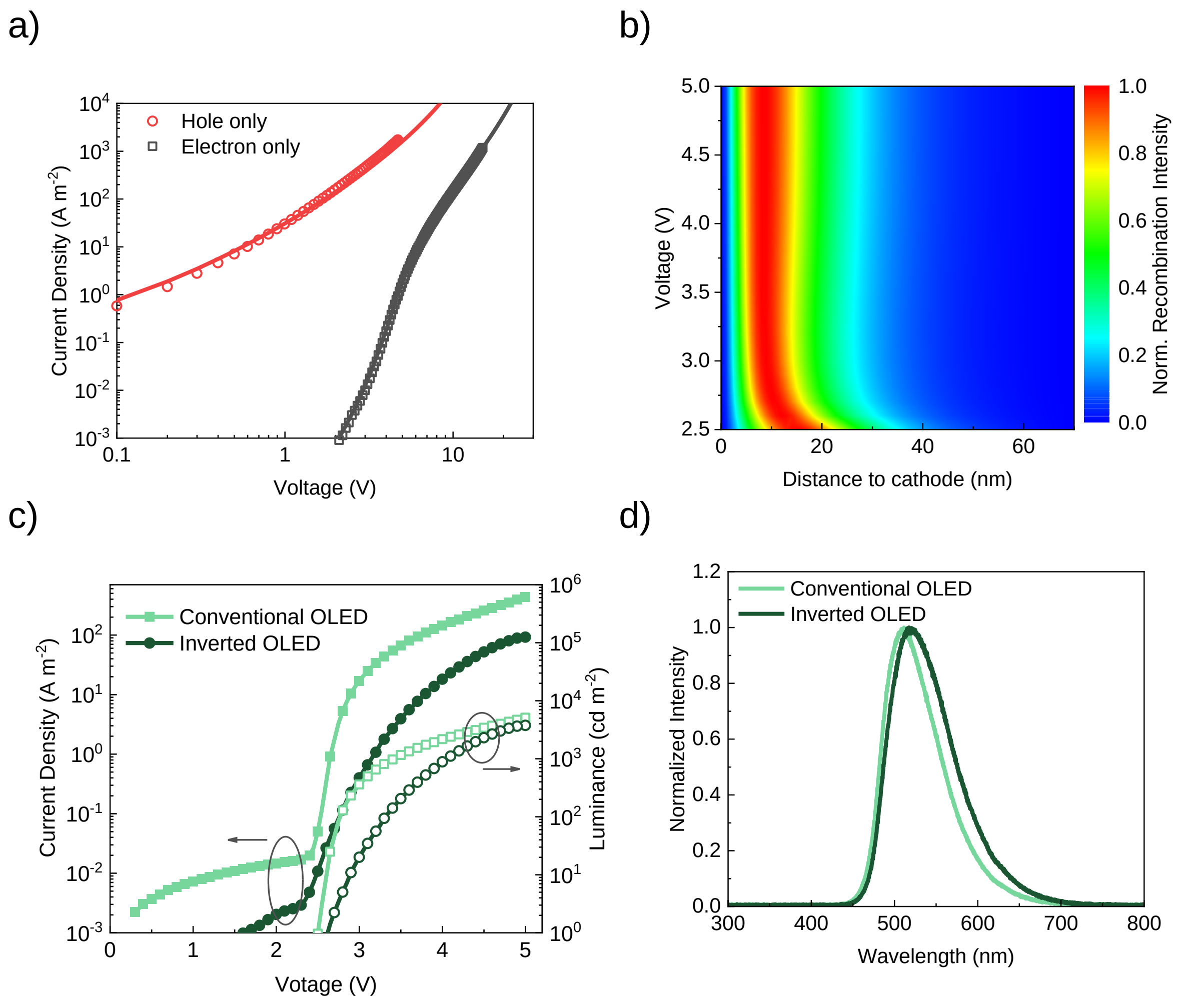

Supplementary Figure 3. Charge transport and simulated recombination profile, optical out-coupling efficiency for DMAC-BP. a) Current density–voltage characteristics of DMAC-BP electron- (110 nm) and hole-only (119 nm) devices (symbols) at 295 K. Solid lines are fitted with a numerical drift-diffusion model. b) Voltage-dependent recombination profile normalized to the total rate for a DMAC-BP OLED with a 70 nm emissive layer. c) Current density–voltage (close symbols) and luminance–voltage (open symbols) characteristics for a DMAC-BP single-layer conventional OLED (70 nm) and inverted OLED (70 nm). d) Electroluminescence spectra of single-layer DMAC-BP conventional and inverted OLEDs.

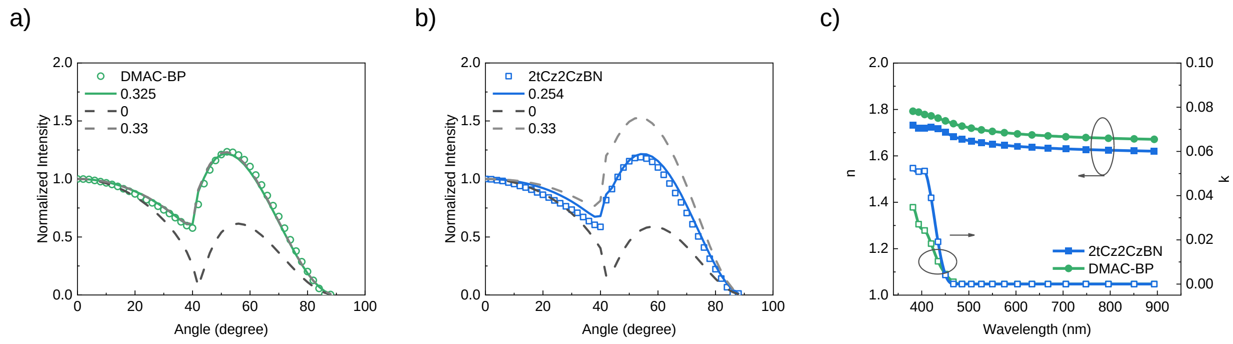

Supplementary Figure 4. Anisotropy factor and refractive index. a) DMAC-BP, b) 2tCz2CzBN determined from the angular-dependent photoluminescence, c) refractive index of 2tCz2CzBN and DMAC-BP determined from ellipsometry.

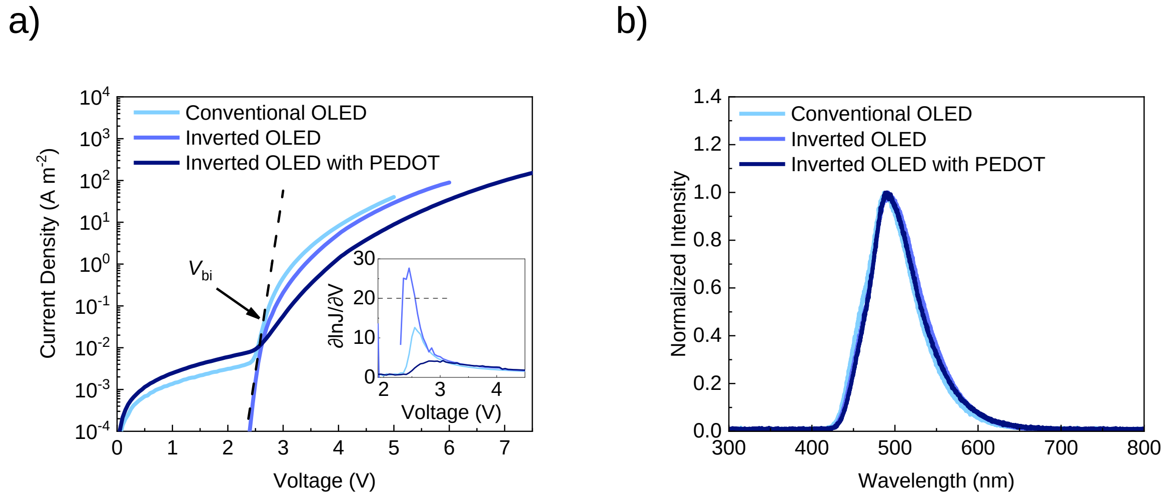

Supplementary Figure 5. Current density-voltage characteristics and EL spectra of blue OLEDs. a) Current density-voltage characteristics of a 2tCz2CzBN single-layer conventional OLED (75 nm), inverted OLED (75 nm) and inverted OLED with an additional PEDOT:PSS layer of 40 nm. From the exponential diffusion-dominated regime (dashed line) the transition to the quadratic drift regime marks the built-in voltage[2]. The inset shows the derivative, from which the built-in voltage can be inferred. The built-in voltage is the same for the inverted and conventional devices. As the built-in voltage is determined by the work function difference between the electrodes, it can be concluded that the n-TFB layer forms a (near-) ohmic contact in the inverted device. This is consistent with the high EQE observed in inverted OLEDs, which would not be possible to achieve for non-ohmic contacts. b) Electroluminescence spectra of single-layer 2tCz2CzBN conventional OLED and inverted OLED.

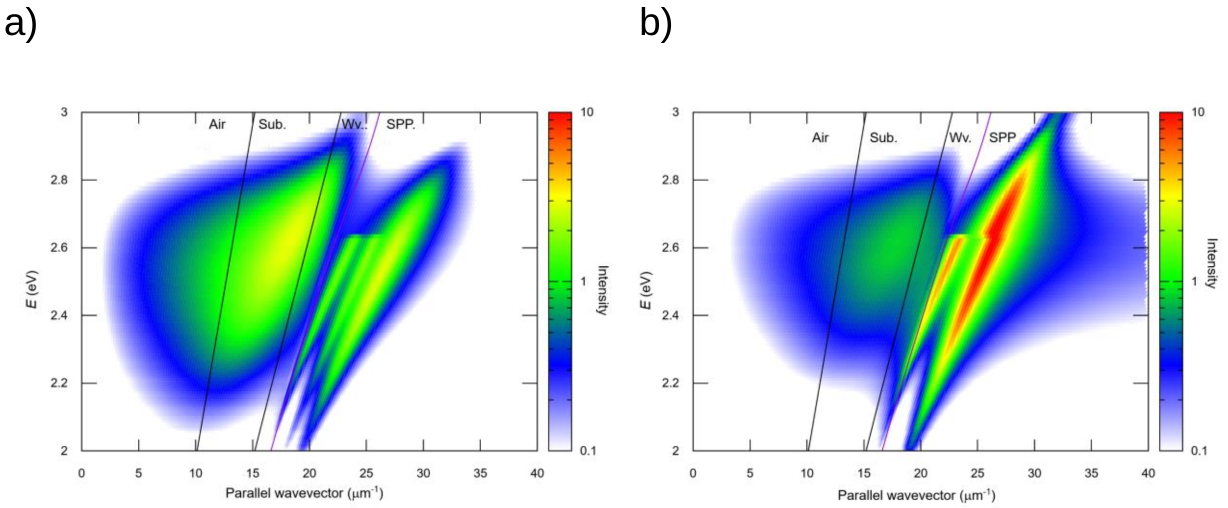

Supplementary Figure 6. Power dissipation for single-layer blue 2tCz2CzBN OLEDs. a) Conventional device structure. b) Inverted device structure. The air, substrate, waveguide, and SPP mode are indicated.

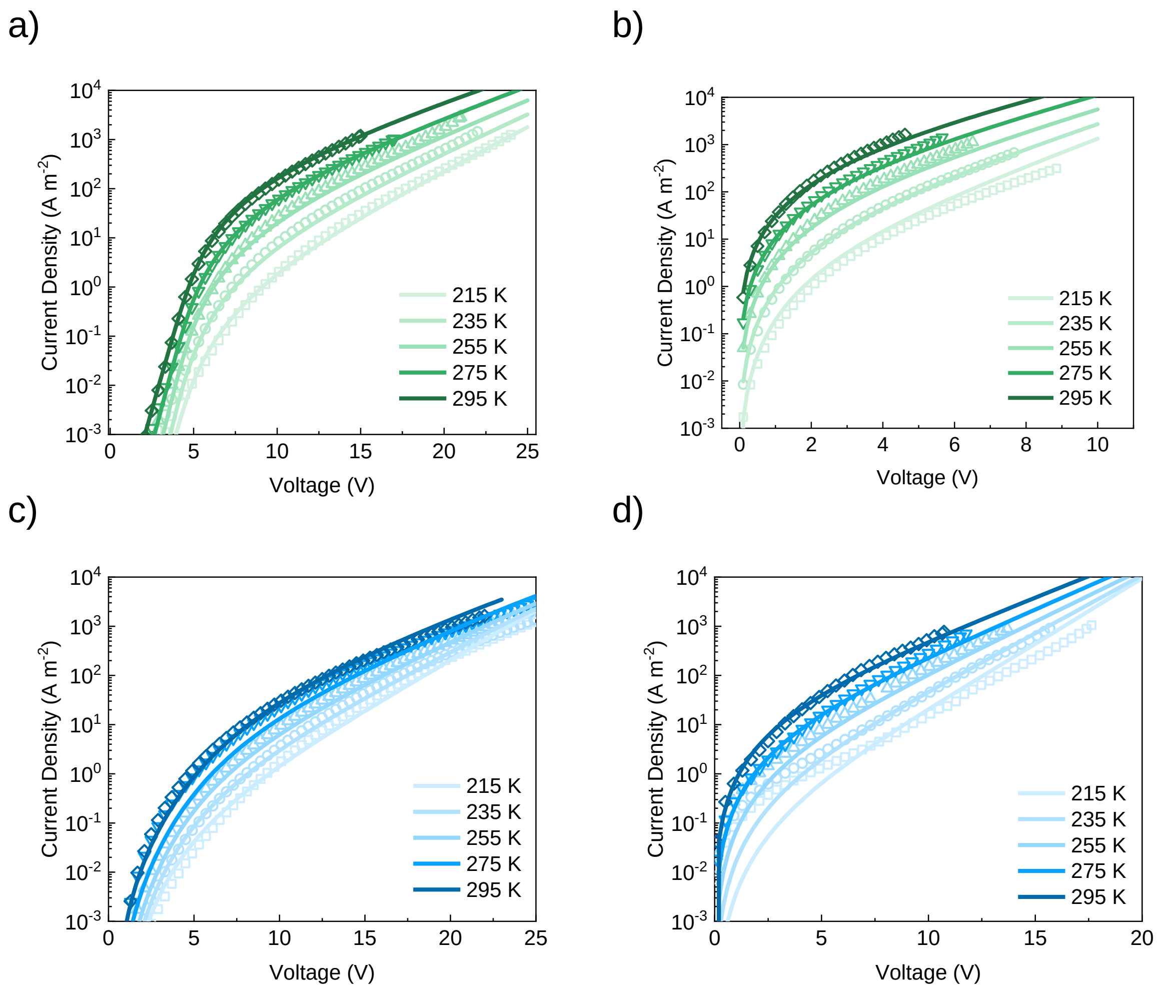

Supplementary Figure 7. Temperature-dependent current density-voltage characteristics. a) DMAC-BP electron-only (110 nm), b) DMAC-BP hole-only (119 nm), c) 2tCz2CzBN electron-only (97 nm), d) 2tCz2CzBN hole-only (108 nm) devices (symbols). Solid lines are fitted with a numerical drift-diffusion model, with parameters as listed in Supplementary Table 2.

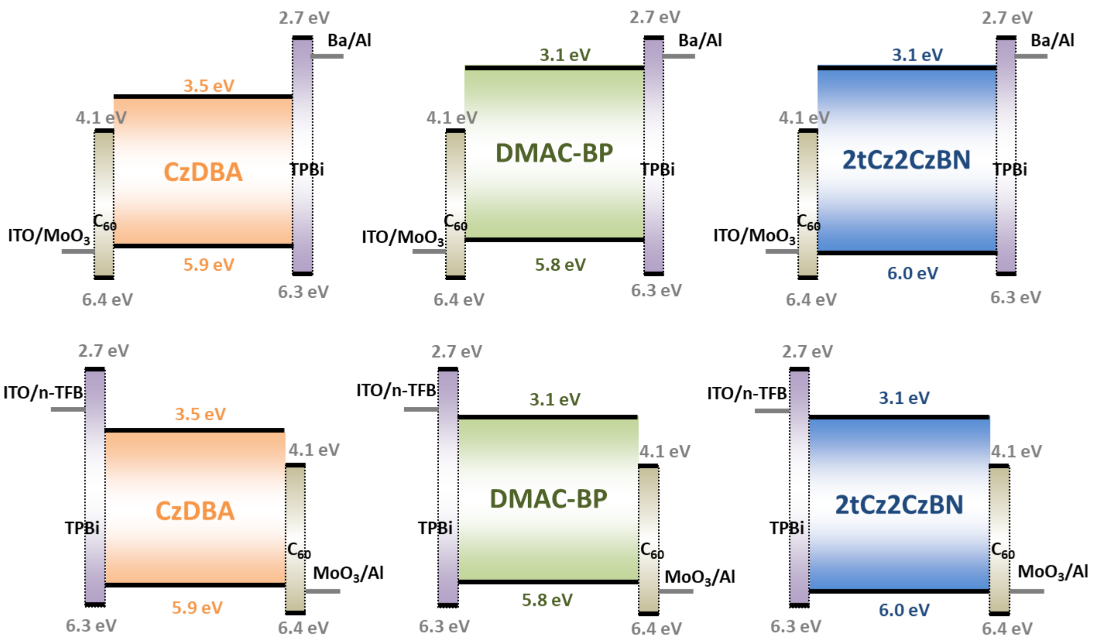

Supplementary Figure 8. Energy diagrams for each emitter in a conventional OLED (top row) and inverted OLED (bottom row) device structure.

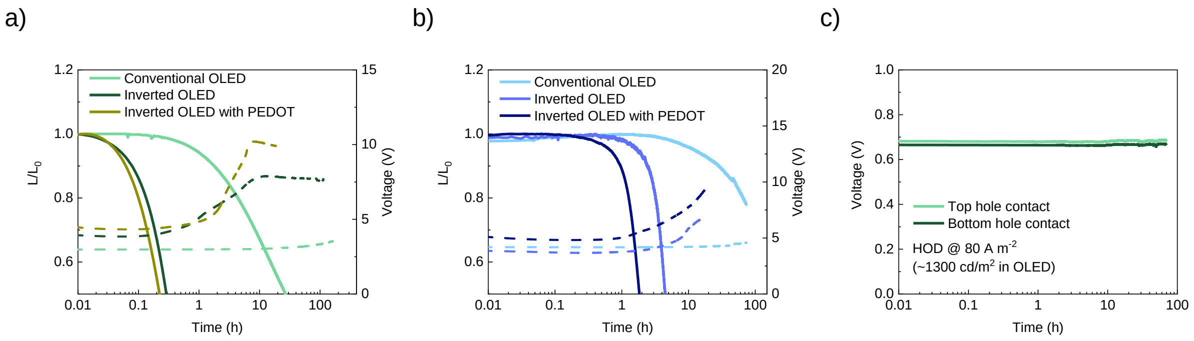

Supplementary Figure 9. Device lifetime. a) DMAC-BP in single-layer conventional (70 nm) and inverted (70 nm) OLEDs stressed at a constant current, at an initial luminance of 1000 cd m^{-2}, b) 2tCz2CzBN in single-layer conventional (85 nm) and inverted (79 nm) OLEDs stressed at 100 cd/m^2, c) Hole-only device (HOD) of DMAC-BP (90 nm), stressed at constant current density (80 A/m^2) which is equivalent to a luminance of 3300 cd m^{-2} for an inverted OLED and 1300 cd m^{-2} for a conventional OLED.

Supplementary Note 1. The lifetime measurements on DMAC-BP indicate that in the conventional device structure, the stability is improved compared to reported multilayer devices[3], despite imbalanced transport. In the inverted devices, the electron-injection layer is limiting the lifetime. This is apparent from the fact

that the voltage remains stable until at least LT_{50}. Since these OLEDs are hole-dominated devices, a stable voltage means that the hole current is unaffected, implying that the degradation is either due to reduced electron injection or reduced electron transport. As the only difference between the conventional and the inverted device is the used electron-injection layer, this is the source of the faster degradation in the inverted OLED. When stressed under constant current, reduced electron injection will hardly affect the driving voltage, since the current is mainly carried by holes from the start. The increase in voltage observed near the end of life is most likely caused by the inability of the electron-injection layer to extract holes, possibly due to dedoping of the n-TFB layer. The experiment on the stressing of the hole-only devices (Supplementary Figure 9c) confirms that the hole-contact (MoO_3/C_{60}) is stable under current stress, both in top and bottom configuration, excluding degradation of this contact as the source of the voltage rise near the end of life in OLEDs.

The inverted devices with an extra PEDOT:PSS layer are similarly stable to the inverted OLEDs without PEDOT:PSS. The observed difference is not considered significant and within the experimental variation of these results.

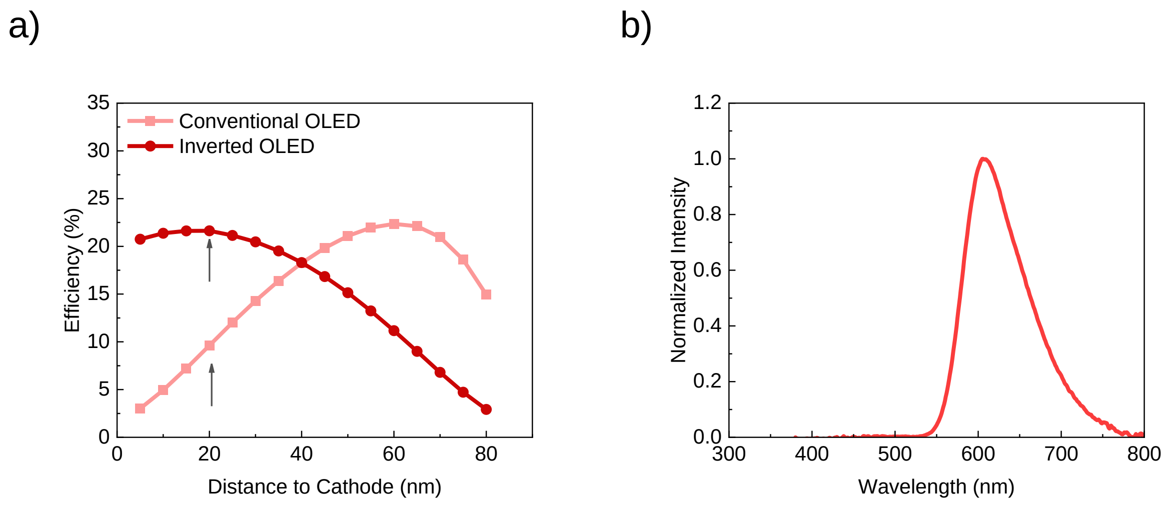

Supplementary Figure 10. a) Simulated optical outcoupling efficiency as a function of the emitter position with respect to the cathode for a red emitter. The arrows indicate the expected position of the recombination zone for an emissive layer with better hole transport than electron transport. b) normalized PL spectrum for the red emitter $Ir(MDQ)_2acac$[4,5]

2. Supplementary Tables

Supplementary Table 1: Device structures

Conventional OLED structure	ITO/ PEDOT:PSS (40 nm)/ MoO_3 (7 nm)/ C_{60} (4 nm)/ emissive layer/ TPBi (4 nm)/ Ba (5 nm)/Al (100 nm)
Inverted OLED structure	ITO/ n-TFB (14 nm)/ TPBi (4 nm)/ emissive layer/ C_{60} (4 nm)/ MoO_3 (10 nm)/Al (100 nm)<br><br>ITO/ PEDOT:PSS (40 nm)/ n-TFB (14 nm)/ TPBi (4 nm)/ emissive layer/ C_{60} (4 nm)/ MoO_3 (10 nm)/Al (100 nm)
Electron only device	Al (30 nm)/ active layer/ TPBi (4 nm)/Ba (5 nm)/Al (100 nm)
Hole only device	ITO/ PEDOT:PSS (40 nm)/ MoO_3 (7 nm)/ C_{60} (4 nm)/ active layer/ C_{60} (4 nm)/ MoO_3 (10 nm)/Al (100 nm)

Supplementary Table 2: Drift-diffusion modeling parameters for single-carrier devices of DMAC-BP and 2tCz2CzBN.

	DMAC-BP<br><br>Hole Only	DMAC-BP<br><br>Electron Only	2tCz2CzBN<br><br>Hole Only	2tCz2CzBN<br><br>Electron Only
Trap density, N_t gauss (× 10^{23} m^{-3})	\	1.6	\	4
Trap depth, E_t (eV)	\	0.8	\	0.6
Width of Gaussian trap distribution, σ_t (eV)	\	0.1	\	0.1
Lattice constant EGDM, a (× 10^{-9} m)	1.7	1.0	1.4	1.0
DOS variance EGDM, σ (eV)	0.144	0.14	0.15	0.15
Mobility at 295 K, μ (× 10^{-9} cm^2/Vs)	428.9	95	5.61	2

3. Supplementary Methods

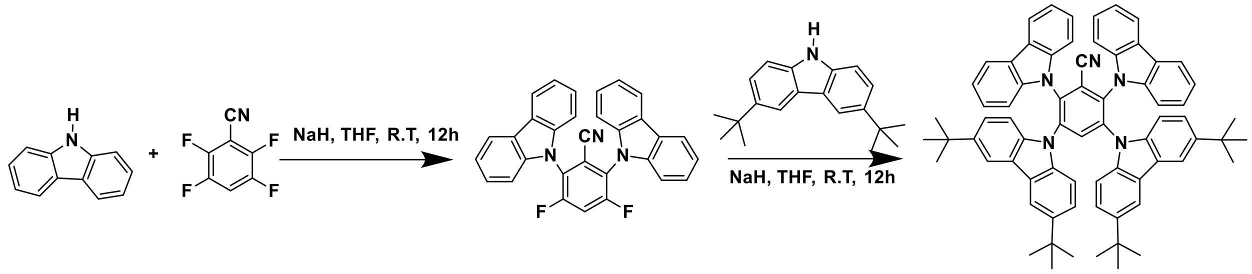

Supplementary Figure 11. Synthesis route for 2tCz2CzBN.

According to literature[6], add carbazole (0.95 g) to a dispersion of sodium hydride (0.135 g) in anhydrous THF (30 ml) at 0 °C. Stir the mixture for 30 minutes. Add 2,3,4,5-4-fluorobenzonitrile (0.5 g)to the mixed solution under argon atmosphere. Stir the reaction mixture at room temperature overnight. Quench the reaction mixture with water. Filter the precipitate. Wash the precipitate with water. Extract the content using chloroform, the crude product was purified by column chromatography on silica gel using n-hexane/methylene chloride. Then proceed to the second step, add 3,6-di-tert-butyl-9H-carbazole (0.7 g) to a dispersion of sodium hydride (0.06 g) in anhydrous THF (30 ml) at 0 °C. Stir the mixture for 30 minutes. Add 2,6-di(9H-carbazol-9-yl)-3,5-difluorobenzonitrile (0.469 g) to the mixed solution under argon atmosphere. Stir the reaction mixture at room temperature overnight. Quench the reaction mixture with water. Filter the precipitate. Wash the precipitate with water. Extract the content using chloroform The crude product was purified by column chromatography on silica gel using n-hexane/methylene chloride (yield is 65%).^{1}H NMR (400 MHz, CD_2Cl_2) δ 8.46 (s, 1H), 7.84 – 7.79 (m, 8H), 7.40 – 7.34 (m, 4H), 7.27 – 7.15 (m, 16H), 1.35 (s, 36H). ^{13}C NMR (101 MHz, CD_2Cl_2) δ 144.55, 139.47, 138.34, 138.06, 137.60, 137.30, 126.09, 124.55, 124.38, 123.77, 121.51, 120.60, 116.54, 110.50, 109.41, 34.91, 31.93. High-resolution mass spectrometry (HRMS) (APCI) *m/z*: calcd for $C_{71}H_{65}N_5$: 987.5240; found： 987.5256 (error: 1.6 ppm).

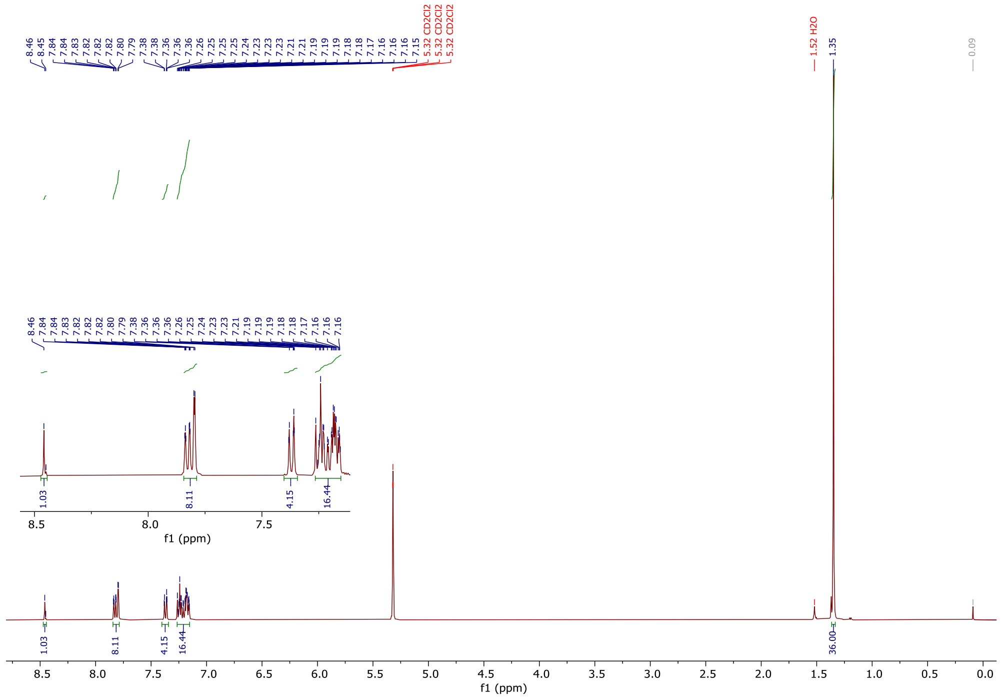

Supplementary Figure 12. **^{1}H NMR spectrum of compound 2tCz2CzBN (400 MHz, CD_2Cl_2).**

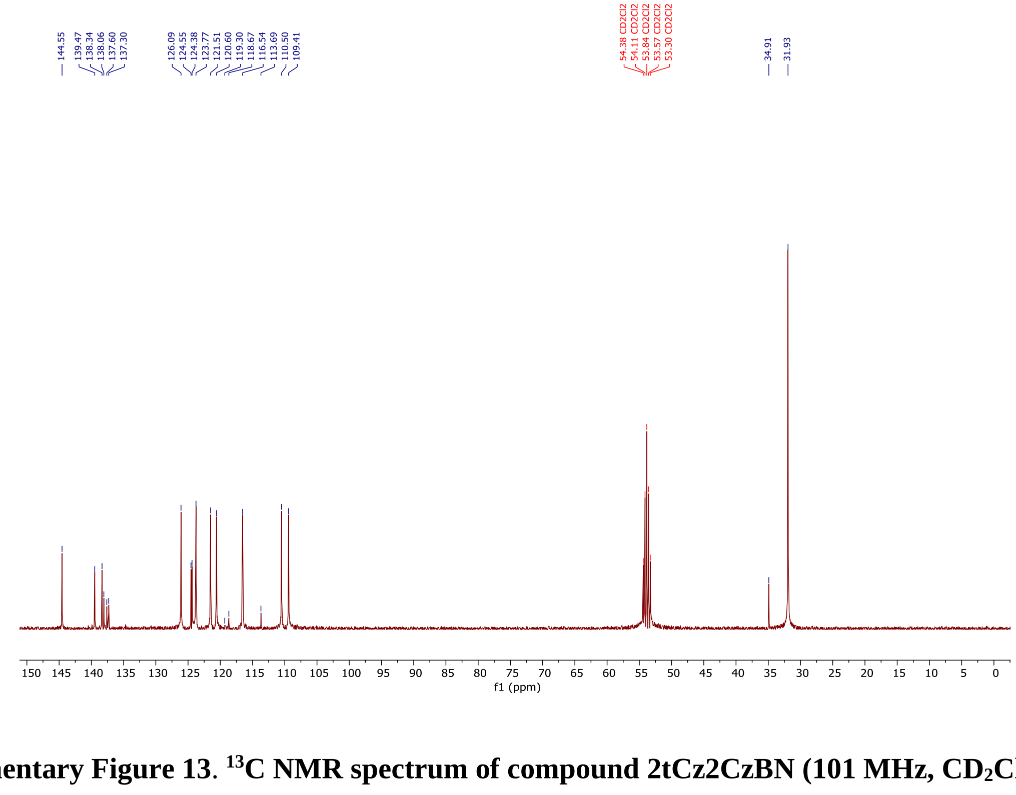

Supplementary Figure 13. **^{13}C NMR spectrum of compound 2tCz2CzBN (101 MHz, CD_2Cl_2).**

4. Supplementary References

1. Forrest, S. R., Bradley, D. D. C. & Thompson, M. E. Measuring the efficiency of organic light-emitting devices. *Adv. Mater.* **15**, 1043–1048 (2003).

2. Wetzelaer, G. A. H. Improved Determination of the Mobility and Built-In Voltage in Asymmetric Single-Carrier Devices. *Phys. Rev. Appl.* **13**, 034069 (2020).

3. Zhang, Q. *et al.* Nearly 100% internal quantum efficiency in undoped electroluminescent devices employing pure organic emitters. *Adv. Mater.* **27**, 2096–2100 (2015).

4. Duan, J. P., Sun, P. P. & Cheng, C. H. New Iridium Complexes as Highly Efficient Orange–Red Emitters in Organic Light-Emitting Diodes. *Adv. Mater.* **15**, 224–228 (2003).

5. Graf, A. *et al.* Correlating the transition dipole moment orientation of phosphorescent emitter molecules in OLEDs with basic material properties. *J. Mater. Chem. C* **2**, 10298–10304 (2014).

6. Zou, S. J. *et al.* High-Performance Nondoped Blue Delayed Fluorescence Organic Light-Emitting Diodes Featuring Low Driving Voltage and High Brightness. *Adv. Sci.* **7**, (2020).
